# Improved contiguity of the threespine stickleback genome using long-read sequencing

**DOI:** 10.1093/g3journal/jkab007

**Published:** 2021-01-23

**Authors:** Shivangi Nath, Daniel E Shaw, Michael A White

**Affiliations:** Department of Genetics, University of Georgia, Athens, GA 30602, USA

**Keywords:** threespine stickleback fish, long-read sequencing, genome assembly, telomere sequence, centromere sequence

## Abstract

While the cost and time for assembling a genome has drastically decreased, it still remains a challenge to assemble a highly contiguous genome. These challenges are rapidly being overcome by the integration of long-read sequencing technologies. Here, we use long-read sequencing to improve the contiguity of the threespine stickleback fish (*Gasterosteus aculeatus*) genome, a prominent genetic model species. Using Pacific Biosciences sequencing, we assembled a highly contiguous genome of a freshwater fish from Paxton Lake. Using contigs from this genome, we were able to fill over 76.7% of the gaps in the existing reference genome assembly, improving contiguity over fivefold. Our gap filling approach was highly accurate, validated by 10X Genomics long-distance linked-reads. In addition to closing a majority of gaps, we were able to assemble segments of telomeres and centromeres throughout the genome. This highlights the power of using long sequencing reads to assemble highly repetitive and difficult to assemble regions of genomes. This latest genome build has been released through a newly designed community genome browser that aims to consolidate the growing number of genomics datasets available for the threespine stickleback fish.

## Introduction

Reference genome assemblies have been invaluable in the discovery of genes, the annotation of regulatory regions, and for providing a scaffold for understanding genetic variation within a species. With the advent of new sequencing technologies and the reduction of cost, there has been a rapid increase in the total number of reference genomes available across taxa. Although it has become much simpler to produce a draft reference assembly, the completion of a high quality, contiguous assembly remains a great challenge. There are many regions within individual genomes that are unassembled. These regions are enriched for highly repetitive sequence that cannot be assembled using sequencing technologies that produce short fragments (Gnerre *et al.* 2011; Nagarajan and Pop 2013). Even the most highly refined genomes, like the human genome still have many gaps, which often are composed of long segmental duplications (Schneider *et al.* 2017).

Long-read sequencing technologies (Oxford Nanopore and Pacific Biosciences) have shown promise in spanning highly repetitive regions of genomes, bridging previously intractable gaps in assemblies to improve overall contiguity. Within the human genome, many highly repetitive regions have been resolved, such as pericentromeres (Vollger *et al.* 2020), complete centromeres (Jain *et al.* 2018b), telomeres (Jain *et al.* 2018a), and the entire major histocompatibility complex (Jain *et al.* 2018a). *De novo* assemblies of highly repetitive Y chromosomes have also become feasible using long-read sequencing (Chang and Larracuente 2019; Peichel *et al.* 2020). Overall, long-read sequencing has enabled chromosome-scale assemblies in multiple species, including many teleost fish (Conte *et al.* 2019; Zhou *et al.* 2019; He *et al.* 2020; Heras *et al.* 2020; Liu *et al.* 2020; Miga *et al.* 2020; Prost *et al.* 2020). It is clear that hybrid assembly approaches incorporating long-read sequencing have greatly improved contiguity of genomes.

Here, we use long-read sequencing to generate a *de novo* Paxton Lake male genome assembly and improve the most recent version of the threespine stickleback reference assembly. The threespine stickleback fish has been an important model system to understand evolution, ecology, physiology, and toxicology (Wootton 1976; Bell and Foster 1994). The identification of the genetic mechanisms underlying many adaptative traits was facilitated by the release of a high-quality reference assembly (Jones *et al.* 2012). This reference assembly was constructed from a single female fish from Bear Paw Lake (Alaska, USA) using paired-end Sanger sequencing of multiple genomic libraries. Contigs were scaffolded to genetic linkage maps, which resulted in 21 chromosome-level scaffolds (400.4 Mb), with 60.7 Mb of unplaced scaffolds. The assembly has undergone several revisions, using high-density genetic linkage maps from multiple populations (Roesti *et al.* 2013; Glazer *et al.* 2015), and a Hi-C proximity-guided assembly from a male from Paxton Lake (Peichel *et al.* 2017). Despite multiple revisions, the latest version of the assembly (v. 4) still contains 13,538 gaps and 20.6 Mb of unplaced scaffolds (Peichel *et al.* 2017). The gaps between contigs in the chromosome scaffolds likely represent repetitive regions or GC-rich regions, which have been shown to be recalcitrant to traditional assembly methods (Benjamini and Speed 2012; Ross *et al.* 2013).

We first generated a *de novo* assembly of a Paxton Lake benthic male threespine stickleback fish. Paxton Lake has been a focal population of threespine stickleback fish to understand the genomic basis of sympatric speciation (McPhail 1992; Hatfield and Schluter 1999; Arnegard *et al.* 2014). Chromosome-level scaffolds of the X and Y from a Paxton lake benthic male were recently assembled using a combination of PacBio sequencing and chromatin conformation capture sequencing (Hi-C) (Peichel *et al.* 2020). We used the remaining assembled autosomal contigs, combined with Hi-C sequencing and optical mapping to produce contiguous chromosome-level autosome scaffolds. We show this assembly is highly colinear with the reference Bear Paw Lake reference genome (v. 4). To improve the contiguity of the existing v. 4 assembly, we used the Paxton Lake contigs to fill gaps in the assembly. We were able to close 76.7% of the gaps, incorporating 13.5% of the previously unplaced scaffolds. Closed gaps were highly accurate, verified through long-distance linked-read information. In addition, we were able to extend sequence of many of the chromosomes into telomeres. This new v. 5 assembly represents a noteworthy improvement, allowing researchers to interrogate many previously inaccessible repetitive regions, and highlights the power of long-read sequencing to substantially improve genome contiguity.

## Materials and methods

### Ethics statement

All procedures using threespine stickleback fish were approved by the University of Georgia Animal Care and Use Committee (protocol A2018 10-003-Y2-A5).

### Paxton Lake benthic male *de novo* assembly

A male Paxton Lake benthic threespine stickleback fish (Texada Island, British Columbia) was previously sequenced using PacBio to approximately 75x coverage (NCBI BioProject database accession PRJNA591630; Peichel *et al.* 2020) and assembled into contigs using Canu (Koren *et al.* 2017). The Canu contigs were previously polished using Arrow (Peichel *et al.* 2020). This assembly had a total of 3593 contigs (N50: 683 kb) from across the genome. X- and Y-linked reads were previously separated from this set of contigs (Peichel *et al.* 2020), leaving a total of 3134 contigs from across the remainder of the genome. Contigs were assembled into scaffolds using Hi-C proximity guided scaffolding, derived from a different male from the Paxton Lake benthic population (NCBI SRA database: PRJNA336561; Peichel *et al.* 2017). Hi-C reads were aligned to the autosome contigs using Juicer (v. 1.5.6) (Durand *et al.* 2016). Autosomes were scaffolded using 3 D-DNA (v. 180114) with—editor repeat coverage 11 (Durand *et al.* 2016; Dudchenko *et al.* 2017; Peichel *et al.* 2020). Accuracy of the scaffolding was verified using BioNano optical maps (Supplementary Table S1). Previously produced optical maps from a different male from the Paxton Lake benthic population (Peichel *et al.* 2017) were aligned to Paxton Lake *de novo* assembly using HybridScaffold.pl within the BionanoSolve software package (v. 3.4). We removed contigs from the Hi-C scaffolds that were not supported by the optical map. A contig was not supported if less than 50% of its length did not overlap with an optical contig. Alignments between the Paxton Lake assembly and the optical contigs were visualized using MapOptics (Burgin *et al*. 2019). Unsupported contigs were removed using a custom Perl script. Collinearity between the Paxton Lake assembly and the v. 4 reference assembly was assessed using nucmer in the MUMmer software package (Kurtz *et al.* 2004). Nucmer was run with default parameters and –mum. Alignments were filtered using delta-filter, retaining alignments with an alignment identity greater than 98% and alignment lengths greater than 4 kb.

### Closing gaps in the reference assembly

Version four of the threespine stickleback reference assembly contains 1263 unplaced contigs (chr. Un) that were narrowed to chromosomes but were not placed into specific gaps (there was a total of 3378 chr. Un contigs: 1263 contigs were narrowed broadly to chromosomes and 2115 could not be localized to any chromosome) (Peichel *et al.* 2017). We used the 1263 chr. Un contigs that were previously narrowed to chromosomes in combination with the Paxton Lake Canu contigs to independently fill the remaining gaps in the reference assembly. To create the v. 5 assembly, we closed gaps in the v. 4 reference assembly using LR_Gapcloser with the parameter -a 1 (Xu *et al.* 2019). We increased the allowed deviation between gap length and the inserted sequence length to provide additional flexibility for gap size that was not inferred accurately in the v. 4 reference assembly. LR_Gapcloser fills existing gaps in the reference assembly by identifying contigs which span a gap completely or partially from either end. Three Paxton Lake Canu contigs caused a reduction in total chromosome size after placement into gaps. Alignment of these contigs to the v. 4 reference assembly shows a small region of homology not linear with the rest of the contig which caused LR_Gapcloser to erroneously ligate the two ends of the gaps (Supplementary Figure S1). We omitted these three contigs from further analysis. We used BLAT (v. 3.5; Kent 2002) to identify which of the 1263 previously narrowed chr. Un contigs from the reference assembly were placed within a gap. We filtered for stringent alignments by only retaining matches where at least 90% of the query length aligned to the assembly and the total aligned region had 2% or less mismatches.

Many chr. Un contigs that were not placed in the v. 4 reference assembly may be represented in the v. 5 assembly if they were contained completely within a Paxton Lake Canu contig (Peichel *et al.* 2020). To test this, we used BLAT to align the 3378 chr. Un contigs to the new v. 5 assembly. We filtered for stringent alignments by only retaining matches where at least 90% of the query length aligned to the assembly and the total aligned region had 2% or less mismatches. Chr. Un contigs that did not align to the assembly were retained as unassembled and concatenated into a single fasta sequence, with each contig separated by 100 N’s (total length: 19.88 Mb with N’s; 19.59 Mb without N’s). Our assembly pipeline is summarized in Supplementary Figure S2.

### Validation of the closed gaps in the v. 5 reference assembly

We verified that gaps were closed correctly in the reference assembly using two approaches. First, we validated that the contigs LR_Gapcloser used to close gaps in the reference assembly were collinear with the Paxton Lake *de novo* assembly. Sequence from the closed gaps from the v. 5 reference assembly were aligned to the Paxton Lake *de novo* assembly using the nucmer utility in MUMMER (v. 4) (Kurtz *et al.* 2004). We also aligned the longer v. 5 contigs, split at gaps that were not closed, to the v. 4 reference assembly. Alignments were stringently filtered for an overall sequence identity greater than 98% and for a minimum length aligned of 4 kb. Second, we used long-distance linked-read sequencing from a female fish from a different freshwater population (Lake Washington, Washington, USA). Segments supported by two independently derived freshwater populations (Paxton Lake and Lake Washington) would suggest gaps closed in the reference assembly (Bear Paw Lake) represent the ancestral state, likely shared among all populations of threespine stickleback fish.

For the linked-read sequencing, we extracted high molecular weight DNA from blood using alkaline lysis. Blood was collected from euthanized fish into 0.85x SSC buffer. The cells were collected by centrifuging for 2 min at 2000 ×g. Pelleted cells were resuspended in 5 ml of 0.85x SSC and 27 µl of 20 µg/ml Proteinase K solution. To lyse the cells, 5 ml of 2x SDS buffer (80 mM EDTA, 100 mM Tris pH 8.0, and 1% SDS) was added to the suspension and the solution was incubated at 55°C for 2 min. After incubation, 10 ml of buffered phenol/chloroform/isoamyl-alcohol was added to the suspension. The suspension was incubated at room temperature under slow rotation for 30 min. The suspension was centrifuged for 1 minute at 2000 ×g at 4°C to separate phases. The aqueous phase was extracted, 10 ml of chloroform was added, and the suspension was rotated for 1 h. The chloroform extraction step was repeated twice. After all extractions, the aqueous phase was separated and mixed with ice cold 100% ethanol and one ml of 3 M sodium-acetate (pH 5.5). The tube was gently inverted until a spool of DNA was observed. The DNA spool was transferred to a 2 ml tube filled with 70% cold ethanol and pelleted at 500 ×g for 2 min. The DNA was allowed to dry at room temperature and resuspended in nuclease free water. Wide bore pipette tips were used for the whole procedure to minimize shearing. The integrity and size of the high molecular weight DNA was verified using a high sensitivity large fragment analysis on a fragment analyzer (Advanced Analytical Technologies, CA, USA). Genomic DNA was size selected to exclude fragments below 50 kb. Linked-read library preparation and sequencing (one Illumina NextSeq 2 × 150 bp high-output flow cell) was conducted by the Georgia Genomics and Bioinformatics Core (University of Georgia, GA, USA). Longranger (v. 2.2.2) was used to trim barcodes from the raw sequences and align the trimmed sequences to the new v. 5 assembly in wgs mode with default parameters (https://github.com/10XGenomics/longranger, last accessed Jan. 29, 2021). The overall alignment rate of linked-reads to the assembly was 84.4%, resulting in a genome-wide mean read depth of 26.1X.

### Assessing the completeness of the v. 5 reference assembly

We assessed the completeness of the v. 5 reference assembly by identifying universal single copy orthologs (BUSCO) in the new assembly, compared to the previous v. 4 assembly (Peichel *et al.* 2017). BUSCO (v. 3.0.2) was run using default parameters, comparing against the Actinopterygii lineage dataset (4584 total single copy orthologs; OrthoDB v. 9) (Simão *et al.* 2015). Actinopterygii was used because threespine stickleback fish are teleosts, which is the largest infraclass of Actinopterygii.

### Identification of telomeric sequences

PacBio long reads with highly repetitive regions are often not assembled into contigs. We identified the telomeric reads by searching for the ancestral metazoan telomeric motif “TTAGGG” or “CCCTAA” (Moyzis *et al.* 1988; Meyne *et al.* 1989; Traut *et al.* 2007) in the raw PacBio reads. We searched for the motif and their respective counts in each read using the awk command-line utility. Reads were considered for further analyses if they had more than 50 occurrences of the motif. These reads were aligned to the v. 5 reference assembly using minimap2 (v. 2.17) (Li 2018) with default parameters to map to PacBio genomic reads (-ax map-pb). Only the primary alignments were retained. Telomeric reads were assigned to a specific chromosome if greater than 10 kb of unique sequence overlapped with one end of a chromosome. Positive telomeric alignments were merged with the v. 5 reference assembly. Repetitive sequence content within telomeres were visualized using the dotplot function in Geneious Prime (v. 2019 1.1) (https://www.geneious.com, last accessed Jan. 29, 2021).

### Identification of centromeric sequences

BLAST+ (blastn; v. 2.7.1) (Camacho *et al.* 2009) was used to identify the 186 bp threespine stickleback CENP-A monomer repeat (Cech and Peichel 2015) in the PacBio Canu assembled contigs. Contigs containing CENP-A repeats were mapped to the new v. 5 repeat masked assembly (see Genome annotation and repeat masking) using minimap2 (Li 2018) with default parameters to map to PacBio genomic reads (-ax map-pb). Contigs were only retained if greater than 10 kb of sequence mapped uniquely to one chromosome side. The number of CENP-A repeats per chromosome were counted using blastn. Dotplots were generated using Geneious Prime (v. 2019 1.1) (https://www.geneious.com, last accessed Jan. 29, 2021).

### Genome annotation and repeat masking

Genome features were lifted over from the previous reference assembly (v. 4) using a hybrid approach. Genome features were first lifted over to the new assembly using the software package flo (Pracana *et al.* 2017). Most of the features were lifted over successfully (98.1%). We used BLAT to lift over the remaining fraction. The sequence for the features not lifted over with flo was extracted from the version four assembly using samtools faidx. These sequences were then aligned to the new assembly using BLAT. For each feature, the longest alignment was chosen.

Many of the closed gaps were not represented in the previous reference assembly (v. 4) and were therefore unannotated. We annotated these regions using the MAKER (v. 3.01.02) genome annotation pipeline (Cantarel *et al.* 2007; Holt and Yandell 2011). These annotations combined evidence from multiple RNA-seq transcriptomes, all predicted Ensembl protein sequences (release 95), and *ab initio* gene predictions from SNAP (v. 2006-07-28) (Korf 2004) and Augustus (v. 3.3.2) (Stanke *et al.* 2006). MAKER was run over three rounds using the RNA-seq transcriptomes and methods previously described (Peichel *et al.* 2020).

Repeats were annotated across the genome using a combination of RepeatModeler (v. 1.0.11) and RepeatMasker (v. 4.0.5) (http://www.repeatmasker.org). Repeats were first modeled using the default parameters of RepeatModeler. Repeats were then annotated and masked using RepeatMasker with default parameters and the custom RepeatModeler database.

We tested for enrichment of repeats and genes in closed gaps throughout the genome by comparing to randomly drawn 10 Mb segments (we placed 9.9 Mb of sequence within gaps; see Results). We also tested for enrichment of repeats and transposable elements in the remainder of the unplaced chr. Un contigs by comparing to randomly drawn 20 Mb segments throughout the assembled genome (19.59 Mb of chr. Un contigs remained unplaced; see Results). We generated a null distributions by randomly drawing 10,000 segments throughout the genome using bedtools (v. 2.29.2) shuffle (Quinlan and Hall 2010). We then used bedtools intersect to count the number of repeats (with option -c for both 10 and 15 Mb segments) as well as the number of bases that overlapped genes (with option -wao for 10 Mb segments) within each random segment.

### Data availability

The 10X Genomics long-distance linked-read sequencing is available on the NCBI BioProject database under accession number PRJNA639125. The v. 5 reference assembly, and the Paxton Lake *de novo* assembly are available on Dryad (doi:10.5061/dryad.qjq2bvqff). The v. 5 reference assembly is also available for download and browsing from the threespine stickleback genome browser (https://stickleback.genetics.uga.edu, last accessed Jan. 29, 2021). All supplemental material has been uploaded to figshare: https://doi.org/10.25387/g3.13435382.

## Results and discussion

### The Paxton Lake genome was assembled into chromosome-level scaffolds

**Figure 1:**
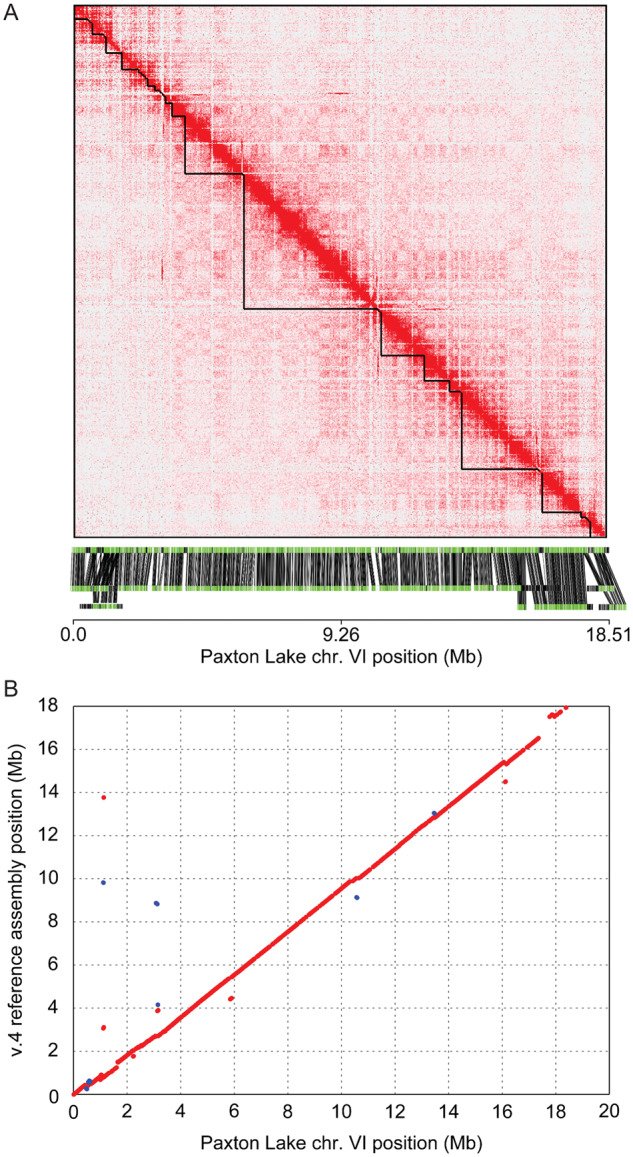
The Paxton Lake *de novo* assembly is collinear with the v. 4 reference assembly. (A) Hi-C chromosome conformation capture sequencing generated a single chromosome VI scaffold. The contact map revealed an enrichment of interactions between contigs that are in close proximity, visualized along the diagonal. Contig boundaries within the scaffold are denoted by black triangles along the diagonal. The corresponding Paxton Lake optical map contigs are concordant with the Hi-C scaffolding. The reference sequence is shown on the top and the optical contigs are shown on the bottom. 97.2% of the chromosome is covered by optical map contigs. (B) Nucleotide alignments between chromosome VI of the two assemblies reveal a syntenic ordering. Blue dots represent small regions of the chromosomes that are in an inverted region relative to the remainder of the alignment (red). The remaining Hi-C scaffold maps, dotplots, and optical alignments are shown in Supplementary Figures S10, S4, and S3, respectively.

A total of 3134 contigs from across the autosomes were assembled into 20 chromosome-level scaffolds. The initial Hi-C proximity guided scaffolded assembly resulted in a total autosome length that was considerably larger than the v. 4 reference assembly (v. 4 reference assembly: 416.97 Mb; Paxton Lake assembly: 473.16 Mb), suggesting there were contigs that were erroneously scaffolded into each chromosome. To explore this, we incorporated long-distance optical mapping contigs (N50: 1.35 Mb) from a different Paxton Lake male fish to refine the assembly (Peichel *et al.* 2017). Consistent with some contigs being erroneously scaffolded, the average percent coverage of the Paxton lake assembly by the aligned optical maps across autosomes was only 87.0% (Figure 1, Supplementary Figure S3). We improved the assembly by removing individual contigs within chromosome scaffolds that were not supported well by the optical alignments (see *Materials and Methods*). After removing contigs, the average percent coverage by aligned optical maps across chromosomes improved (95.2%). In addition, the total length of autosomes of the Paxton Lake *de novo* assembly more closely matched the v. 4 reference assembly (v. 4 reference assembly: 416.97 Mb; Paxton Lake assembly: 427.45 Mb; Table 1). With the addition of the previously assembled Paxton Lake X chromosome sequence (chr. XIX; Peichel *et al.* 2020), the total genome length was 448.50 Mb (Table 1).

**Table 1:** Chromosomal lengths (bp) of the Paxton Lake assembly and the v. 4 reference assembly

Chr.	v. 4 reference assembly	Paxton Lake assembly
**I**	29,714,595	30,291,332
**II**	23,752,435	24,322,974
**III**	17,815,537	17,075,251
**IV**	34,244,925	35,558,100
**V**	15,579,443	16,703,751
**VI**	18,862,055	18,511,259
**VII**	30,864,241	33,510,017
**VIII**	20,606,801	22,749,572
**IX**	20,880,404	21,858,600
**X**	18,035,923	17,341,068
**XI**	17,683,359	18,065,382
**XII**	20,811,783	20,111,693
**XIII**	20,800,062	22,032,464
**XIV**	16,179,395	15,892,220
**XV**	17,375,354	18,397,630
**XVI**	19,558,478	18,726,499
**XVII**	20,254,007	21,751,973
**XVIII**	15,989,023	15,795,861
**XX**	20,484,364	21,645,813
**XXI**	17,480,265	17,105,228
**Autosome total**	**416,972,449**	**427,446,687**
**XIX**	20,618,466	20,783,580
**Genome total**	**437,590,915**	**448,230,267**

Genome wide, the Paxton Lake assembly was highly collinear with the v. 4 reference assembly (Supplementary Figure S4). However, unlike the v. 4 reference assembly, the Paxton Lake assembly was more contiguous. The Paxton Lake assembly had longer contigs (N50: 1.25 Mb) and only 1484 gaps across the autosomes, whereas the v. 4 reference assembly had a total of 12,960 autosomal gaps between shorter contigs (N50: 91.7 kb). Across the genome, we detected 16 small inversions between the Paxton Lake assembly and the v. 4 reference assembly (Supplementary Figure S4; Table S2). We compared these breakpoints with the aligned optical map contigs to identify whether these were true inversions within the Paxton Lake population. An inversion would be supported if it was embedded within an optical mapping contig that was completely collinear with the assembly. All inversion breakpoints either fell at the edge of an optical contig or were not located within an optical contig, suggesting these may reflect assembly errors in the Paxton Lake assembly. Additional refinement will be necessary to determine if these small inversions reflect assembly errors or true structural variants within the Paxton Lake population.

### A majority of gaps were closed across the threespine stickleback reference assembly

Since the *de novo* Paxton Lake assembly was more contiguous than the v. 4 reference assembly, we used the Paxton Lake Canu contigs in conjunction with the 1263 v. 4 chr. Un contigs that had been narrowed to chromosomes to attempt to close the 13,538 gaps in the v. 4 reference assembly. Using LR_Gapcloser we closed 10,394 of the gaps (76.8%), leaving only 3144 gaps in the v. 5 assembly (Xu *et al.* 2019) (Supplementary Files S1, S2, Figure S5). In addition to the fully closed gaps, 146 gaps were partially closed. A total of 9,928,283 bases were added to gaps in the assembly. This resulted in an overall greater contiguity of the genome, with a 5.57-fold greater N50 contig length within scaffolds compared to the previous reference assembly (v. 5 N50: 510.8 kb; v. 4 N50: 91.7 kb) (Table 2).

**Table 2:** Improved contiguity of the threespine stickleback genome

	v. 5 assembly	v.4 assembly
**Assembly size (without Ns and chrUn)**	448.67 MB	441.86 MB
**Number of gaps**	3,144	13,538
**L50**	233	1,291
**L90**	983	5,378
**N50**	510.82 kb	91.68 kb
**N90**	94.65 kb	18.17 kb

Genome contiguity and annotation completeness is often assessed by BUSCO (Benchmarking Universal Single Copy Orthologs) statistics (Waterhouse *et al.* 2018). We determined if the additional sequence in the v. 5 reference assembly contained coding sequence that improved overall BUSCO metrics. Of the 3640 genes within the database, we found a total of 3521 BUSCO genes in the assembly (96.7%). This represented an increase of 99 genes compared to the previous assembly. In addition, the total number of fragmented BUSCO genes decreased to 14, compared to 108 in the v. 4 reference assembly (Supplementary Table S3).

Of the 3378 chr. Un contigs from the v. 4 reference assembly, we determined how many were represented in the closed gaps of the new v. 5 reference assembly. Of the 3378 contigs, 457 contigs were placed within gaps (13.5%). The previous assembly used a Hi-C-based proximity-guided assembly method that was able to narrow some of the chr. Un contigs (1263) to chromosomes, but was not able to place these contigs into specific gaps (Peichel *et al.* 2017). We used this information to verify whether our contig placement was corroborated by the Hi-C sequencing. Of the 1263 previously narrowed chr. Un contigs, we placed 90 of into gaps. A majority of these contigs (80.0%) fell within the same chromosome they were assigned to previously by the Hi-C proximity-guided method. This high concordance further confirms the reliability of our methodology and closure of the gaps.

Across all closed gaps, we added 9.93 Mb of sequence to the genome. 1.13 Mb of this newly added sequence was from chr. Un contigs previously sequenced, but not placed in chromosomes. The remaining 8.80 Mb represented new regions from the long-read sequencing. Many of the gaps in the genome likely represent highly repetitive regions that are challenging to assemble. We compared the repetitive sequence content between the 9.93 Mb of newly added sequence and the remainder of the genome. Indeed, we found newly closed gaps are enriched for repeat sequences (simple and interspersed repeats; 10,000 permutations; *P *<* *0.001; Supplementary Figure S6). Overall, 17.4% of newly added bases contained repetitive DNA compared to 13.5% in the remainder of the genome. Across all newly added gap sequence, we found an overlap with a total of 1602 protein coding genes in v. 5. 1280 of such the genes that were fragmented in v. 4 are now contiguous in v. 5 (Supplemental File S3). The newly placed regions overall exhibit a slightly lower density of coding sequence compared to the remainder of the genome (Supplementary Figure S7; 10,000 permutations; *P* < 0.083). Only 7.9% of the closed gap bases were contained within coding regions. Across the remainder of the genome, 28.3% of bases in the v. 5 reference assembly were contained within coding regions. Combined, our results suggest the highly repetitive nature of the sequence contained within these gaps may have prevented assembly of these regions.

Although we closed a majority of gaps in the assembly, we were unable to determine where 2921 of the chr. Un contigs belonged in the assembly (total length: 19.59 Mb). One possibility why we were unable to place these contigs is that they contain a greater proportion of repetitive sequence. Consistent with this, the unplaced contigs were highly enriched for Gypsy retrotransposons compared to the placed chr. Un contigs (*P *<* *0.001; Supplementary Figure S6). 9.7% of the bases in unplaced contigs overlapped with Gypsy elements compared to 1.3% of the bases across the remainder of the genome. It is also possible that these contigs represent segments of the genome outside of gaps that are mis-assembled. Our method only focused on closing gaps between contigs. Additional work will be necessary to determine whether these contigs integrate elsewhere in the genome. Assembly of these contigs may be facilitated by using additional *de novo* genome assemblies from other populations of threespine stickleback fish (Berner *et al.* 2019).

### Gap closing was validated by long-distance linked reads and collinear alignments with the Paxton Lake assembly

**Figure 2:**
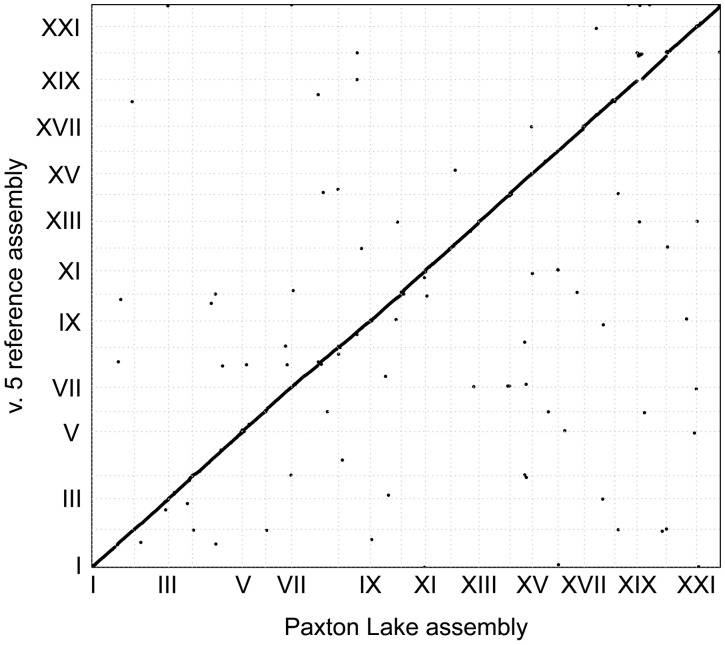
Gaps closed in the v. 5 reference assembly are collinear with the Paxton Lake assembly. Gaps were closed in the v. 5 reference assembly using the unassembled Paxton Lake contigs and the chr. Un contigs from the v. 4 reference assembly. Only 0.01% of the closed gaps aligned to different chromosomes in the Paxton Lake assembly, either reflecting true structural variation or assembly error in either of the populations.

We aligned all gap sequences that were closed in the v. 5 assembly by LR_Gapcloser back to the *de novo* assembled Paxton Lake assembly to see if they were independently placed in the same chromosomal position by the two approaches. Of the 10,394 gaps closed in the v. 5 assembly, we were able to align 8552 (82.3%) back to the Paxton Lake assembly (Figure 2). The missing 1842 gap contigs that were placed in the v. 5 reference assembly by LR_Gapcloser were not assembled In Paxton Lake using the *de novo* assembly pipeline. Of the 8552 aligned gaps only 78 (0.01%) aligned to different chromosomes in the two assemblies. The remaining contigs exhibited highly collinear placements in the two assemblies (Figure 2), supporting accurate gap closing in the v. 5 reference genome.

**Figure 3:**
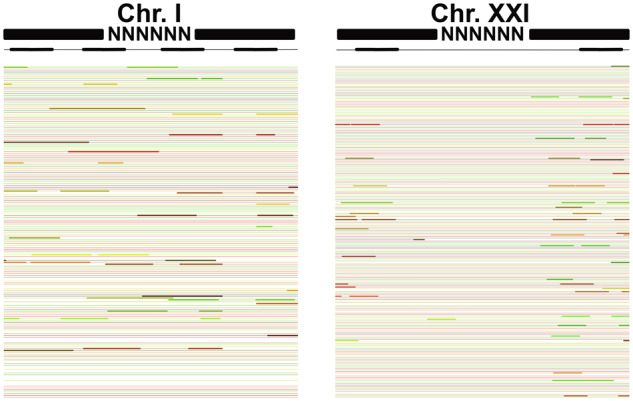
10X Genomics linked-reads validate most of the closed gaps. 99.7% of closed gaps exhibit linked-read alignments throughout the gap region, indicating a correctly closed gap (*e.g.*, Chr. I: 192,954–193,294 bp with flanking region). 0.03% of gaps were not validated by the linked-read sequencing. In these regions, alignments of the linked-reads only occur outside of the gap (*e.g.*, Chr. XXI: 9,436,991–9,437,593 bp with flanking region). A representative schematic outlining how the linked-reads should align is shown in black. The actual aligned linked-reads are shown by bolded color lines. Thin lines indicate gaps between the linked-reads. Average read depth of linked-reads across the genome was 26.1X. A subset of reads aligning is shown here for simplicity.

We used long-distance linked-reads to also validate placement of the new sequence within gaps. Linked-read molecules that support closure of a gap would exhibit aligned short-reads throughout the closed gap, whereas linked-read molecules that do not support closure of a gap would have aligned short-reads outside of the gap, but a lack of alignment within the gap (Figure 3). Similar to the alignment between the Paxton Lake and v. 5 reference assembly, the gap closures were highly supported by the linked-read alignments. We only observed 36 gaps (0.3%) that were not supported by linked-reads (*i.e.*, a lack of short-read alignments over the newly added sequence). The remainder of the 10,394 gaps in this analysis that were closed (99.7%) were supported by the long-distance linked-read dataset (Figure 3). We did not remove the small percentage of gaps that were not supported by the linked-read molecules or with alignment to the Paxton Lake assembly. It is possible this small number of closed gaps reflected true structural variation between the different populations. We therefore included them in the final assembly.

### Telomere repeats and centromere repeats were identified within PacBio long reads

**Figure 4:**
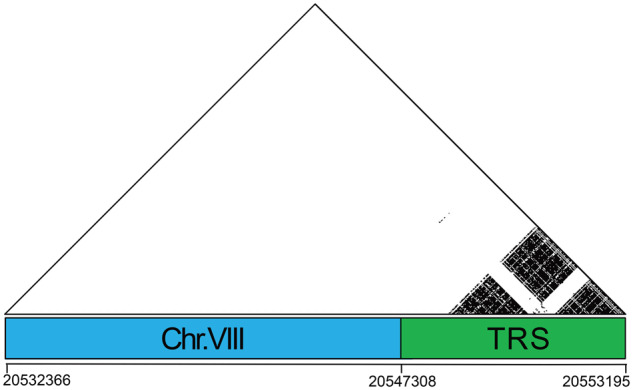
Telomeres exhibit a high density of the conserved metazoan telomere motif. Dots represent 100% sequence identity between matching windows of 15 bp. The blue box represents the end of chromosome VIII where the long read aligns uniquely. The green box denotes a segment rich with telomeric repeat sequence (TRS). The remaining telomeres are shown in Supplementary Figure S8.

The telomeres of threespine stickleback fish contain a tandemly repeated G-rich hexanucleotide repeat that is conserved across metazoans (Moyzis *et al.* 1988; Meyne *et al.* 1989; Traut *et al.* 2007; Ocalewicz 2013). Although DNA probes targeting these repeats clearly hybridize at the ends of all chromosomes in threespine stickleback fish, the underlying sequence of these regions is missing from the reference assembly. We therefore searched for the ancestral metazoan telomeric motif “TTAGGG” or “CCCTAA” in the raw PacBio reads to identify putative telomere caps (Ocalewicz *et al.* 2011). We identified 3525 PacBio reads that contained telomere motifs. Seven of these reads contained enough unique sequence to align to the end of individual chromosomes (chromosomes IV, VII, VIII, X, XIV, XV, and XVII). These reads showed an abundance of the ancestral metazoan telomeric motif at one end with little to no higher order structure (Figure 4; Supplementary Figure S8). The telomeric motif was repeated 114–492 times throughout the sequence on different chromosomes.

**Figure 5:**
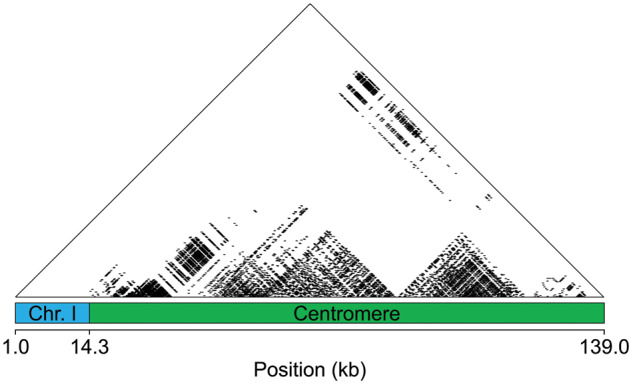
Centromeres display higher order repeat structure. On chromosome I, the centromere contig contains 673 copies of a 186 bp monomer repeat. Sequence identity between repeats is depicted by black dots matching windows of 300 bp with 100% sequence identity. The blue region denotes the side of chromosome I (20,330,007–20,344,665 bp) with unique sequence that aligns to the periphery of the centromere. The green region is the newly aligned centromere contig. The other side of the centromere did not align to the other arm of chromosome I. The remaining centromeres are shown in Supplementary Figure S9.

We also searched for centromere repeats within the PacBio assembled contigs. We identified the core 186 bp CENP-A repeat (Cech and Peichel 2015) within 91 contigs (the length of repetitive DNA among contigs ranges from 12.61 to 125.17 kb). Forty-eight of these contigs contained enough unique sequence to align to all 21 chromosomes (Figure 5; Supplemental Files S4 and S5). 11 chromosomes had centromere contigs that mapped to both sides of the gap, 9 chromosomes had a centromere contig that mapped to only one side of the centromere, and one contig contained a full centromere sequence, spanning the entire gap (chromosome IX). Interestingly, on many of the chromosomes, the repeat length was long enough to discern clear higher order repeat structure (Figure 5; Supplementary Figure S9). Our results are similar to the variability in higher order repeat structure among the autosomes and X chromosome of humans (Willard 1985; Willard *et al.* 1986; Alexandrov *et al.* 1993; Shepelev *et al.* 2015; Hartley and O'Neill 2019). We detected multiple contigs mapping to either side of the centromere gap for all chromosomes (Supplemental File S4), indicating the male fish used for sequencing is likely heterozygous for centromeric arrays. This is consistent with high polymorphism of centromere arrays observed within other species (Willard *et al.* 1986; Devilee *et al.* 1988; Wevrick and Willard 1989; Mahtani and Willard 1990; Greig *et al.* 1991).

Y chromosomes in mammals have also been documented to have highly variable centromeric repeats that are divergent from their counterparts across the remainder of the genome (Wolfe *et al.* 1985; Pertile *et al.* 2009; Miga *et al.* 2014). Assembly of segments of the threespine stickleback Y chromosome centromere (Peichel *et al.* 2020) revealed an alpha satellite monomer repeat that was divergent from the consensus monomeric repeat identified from the remainder of the genome (Cech and Peichel 2015). With the assembly of larger tracks of centromeric sequence from the autosomes and the X chromosome, we now show the Y chromosome centromere is also divergent from the remainder of the genome at the level of higher order repeats (Peichel *et al.* 2020), matching other rapidly evolving Y chromosomes. Although our assembly has uncovered a large fraction of the centromeric sequence for each chromosome, we were unable to assemble complete centromere sequences outside of the 46.5 kb centromere of chromosome IX. It therefore remains unknown how centromere length varies throughout the threespine stickleback genome. Complete characterization of the centromeric repetitive arrays will be aided by future sequencing of ultra-long reads (Jain *et al.* 2018b; Miga *et al.* 2020).

## Conclusions

By using long-read sequencing we were able to substantially improve the overall contiguity of the threespine stickleback reference genome assembly, increasing the N50 length of contigs over fivefold. Our assembly also highlights the power of using long-read sequencing technologies to assemble previously inaccessible regions of the genome, like centromeres and telomeres. We have released this assembly through a new threespine stickleback fish community genome browser (https://stickleback.genetics.uga.edu, last accessed Jan. 29, 2021). The v. 5 reference assembly and the Paxton Lake *de novo* assembly will be useful additions to the rapidly expanding functional genomics toolkit available in threespine stickleback fish.
